# Triptans utilization in Italian population: A real-life study in community pharmacies

**DOI:** 10.1371/journal.pone.0291323

**Published:** 2023-09-08

**Authors:** Francesca Baratta, Gianni Allais, Roberto Gnavi, Cecilia Scarinzi, Lorenzo Ravetto Enri, Sara Rolando, Teresa Spadea, Giuseppe Costa, Chiara Benedetto, Massimo Mana, Mario Giaccone, Andrea Mandelli, Gian Camillo Manzoni, Gennaro Bussone, Paola Brusa

**Affiliations:** 1 Department of Drug Science and Technology, University of Turin, Turin, Italy; 2 Department of Surgical Sciences, Women’s Headache Center, University of Turin, Turin, Italy; 3 FI.CEF Onlus, Italian Headache Foundation, Milan, Italy; 4 Epidemiology Unit, ASL TO3, Grugliasco (Turin), Italy; 5 ATF Informatics, Cuneo, Italy; 6 Order of Pharmacists of Turin, Turin, Italy; 7 FOFI, Federation of the Orders of Italian Pharmacists, Rome, Italy; Global Health Neurology Lab / NSW Brain Clot Bank, NSW Health Pathology / Liverpool Hospital and South West Sydney Local Health District / Neurovascular Imaging Lab, Clinical Sciences Stream, Ingham Institute, AUSTRALIA

## Abstract

The term Headache Disorders (HD) refers to a number of nervous system pathologies characterised by recurrent headaches. Despite the serious impact HD have on the health system, society, and the economy, these are an underestimated, underdiagnosed, and, hence, undertreated phenomenon. Triptans are the first-line therapy for the acute treatment of moderate to severe migraine but their utilization is still inadequate, perhaps also because in Italy no triptan can be bought without a medical prescription. In this article, the data from a 2016–2017 study has been further analysed with the aim of evaluating any associations between the use of triptans and the other series of variables identified in the questionnaire. This further analysis has been connected to the role that community pharmacies could play on this issue. The questionnaire was administered to 4,424 pharmacy users by 610 purposely trained pharmacists working in 514 pharmacies. The survey was carried out in 19 of the 20 Italian regions. The data shows that only 25% of patients suffering from HDs are prescribed triptans. Older patients, those with definite migraines, and those with a chronic disorder resort more frequently to this class of pharmaceuticals, as do those patients in care at a specialist headache centre. The multivariable analysis also confirmed these results. Our study, which performed a direct detection, in real life, on patients requesting pharmacological treatment for a migraine headache, therefore confirmed the need to investigate the reasons behind the low use and prescription of triptans in the Italian population. Moreover, any future studies should take advantage of community pharmacies, plan actions that would allow a series of evaluations over time of the requirements of migraineurs, and establish a process to put these patients under the care of the pharmacy to ensure adherence to therapy.

## Introduction

The term Headache Disorders (HD) refers to a number of nervous system pathologies characterised by recurrent headaches that are broadly classified as primary headache (PH), or secondary headache [1]. The former includes disorders such as migraine headache, tension-type headache, and cluster headache [2], while the latter, in which the pain is a symptom of another condition, includes one of the most common secondary disorders, namely Medication-Overuse Headache (MOH) [3].

Collectively, these disorders have a high prevalence: migraine headaches, for example, affect more than one billion people worldwide. Moreover, approximately 40% of people worldwide have a strong and disabling migraine episode at least once a year [2, 4].

The various types of primary headache (PH) have different characteristics, prevalence, and physio-pathologies; although non-fatal, PH may be extremely debilitating and the quality of life of subjects with recurring headaches is undoubtedly worsened [5]. In 2019, PHs were the main cause cited for 46.6 million Years Lived with Disability (YLDs) globally, that is, 5.4% of the total YLDs, and migraines accounted for 88.2% of this figure [6].

Given that chronic headaches lead to high levels of disability, and poor health [2], not only do they represent a health or social problem, but also have an economic impact due to the loss of working days and productivity. A study conducted in Spain in 2018 showed that a reduction of one day with headache per month saved €744.14 per patient/year in the case of episodic migraine, and €663.20 per patient/year in the case of chronic migraine. A 50% reduction in the number of days with migraine could result in a saving of €2,232.44 per patient/year in the case of episodic migraine, and €6,631.99 per patient/year in the case of chronic migraine [7].

Despite the serious impact HD have on the health system, society, and the economy, they continue to be a phenomenon that is underestimated, underdiagnosed, and, hence, undertreated [2].

In Italy, there are about 19,000 community pharmacies: an average of one for every 3,100 people [8]. Thanks to their distribution throughout the territory, and their extended opening hours, community pharmacies are often the first point of contact with the national health system in the area. For this reason, the community pharmacy is in a strategically important position to identify the health requirements of the local population, and inform subjects unaware of their particular pathologies such as, for example, migraine [9–18]. Between 2016 to 2017, following on from a previous study in the Piedmont region [19], a national survey on migraine was carried out in community pharmacies. The investigation was conducted by means of a questionnaire administered by a trained pharmacist to subjects entering the pharmacy requesting medicine for the self-treatment of headache [9, 14]. The results revealed that a high number of Italian community pharmacies users were migraineurs. In addition, the survey threw up an alarming figure: approximately one third of headache sufferers, and a half of migraineurs did not perceive their disorder as a pathology and did not treat it correctly. Furthermore, only 38% of subjects suffering from a definite migraine were being treated with triptan to block the attack [9].

Thus, the community pharmacy has proven itself to be the ideal place to address the issue of PHs [9].

Triptans are the first-line therapy for the acute treatment of moderate to severe migraine, but their utilization is still inadequate. Fisher et al., indeed report that almost three quarters of patients admitted for a preliminary observation at the Headache Outpatient Clinic had never taken triptans before [20–22]. Despite the specificity and usefulness of triptans, which have been available on the market for over 25 years, also in our study we found that only one third of severe migraineurs use this class of medication.

Hence, in this article, the data from the 2016–2017 study has been further analysed with the aim of evaluating any associations between the use of triptans and the other variables identified in the questionnaire.

## Materials and methods

The study was designed to be a cross-sectional study. The data was gathered by administering a questionnaire drawn up by experts and based on scientific literature [9].

The questionnaire was administered to pharmacy users by purposely trained pharmacists. Pharmacists were selected on a voluntary basis and did not receive any payment for their participation in the survey. The survey was designed in order to ensure that the data were collected as homogeneously as possible, therefore the pharmacist had to attend a national online training course and only those who passed the final test were enrolled in the project [9, 14].

The survey was carried out in 19 of the 20 Italian regions, and involved 610 pharmacists working in 514 pharmacies.

Briefly, the questionnaire (available as S1 Questionnaire) covered the following points: the socio-economic background of the recruited subjects; the ID Migraine screener test (ID-MS) [23, 24]; the frequency of attacks, the usual therapy to treat an attack and the healthcare professional, if any, responsible for the management of the subject’s headache.

Participation in the survey followed a non-random design; all customers entering the participating pharmacies who requested a self-treatment medication for a headache episode were informed about the aims of the study and invited to take part. Enrolment continued from 01/06/2016 to 31/01/2017, each month enrolling the first 5 users who accepted to participate.

### Outcome definition and recording

The intake of triptans was the outcome of interest.

ID-migraine was categorized in four levels (“Definite migraine” (3/3 positive answers), “Probable migraine” (2/3 positive answers), “Unlikely migraine” (1/3 positive answers), “Other headaches” (0/3 positive answers) [9].

Age at enrolment was categorized in three levels: less than 30 years old, 30–54 years, and more than 54 years old. Educational level was codified using the standardized “International Standard Classification of Education” [25], combining no educational level and primary school in ISCED0-1. Secondary school and diploma in ISCED2-3, and degree in ISCED4-6. The variable “number of days with headache in the last three months” was categorized in two ways: as four variable levels (0–10. 11–29. 30–60 and 60–90 days) and as a dichotomous variable (0–44 and 45–90 days). The municipality of residence was categorized in two geographical areas (north and centre-south of Italy).

People who declared to take pain medications for more than 10 days a month for three consecutive months were classified as Medication Over-users (MOs). The term pain medication refers to the medicine usually used by the respondent to relieve the headache symptoms.

More details on the protocol of the survey are reported elsewhere [14].

### Statistical methods

We analysed the association of socio-demographic and clinical information with the use of triptans, either as a single prescription or in association with non-steroidal anti-inflammatory drugs (NSAIDs), by means of relative frequencies and corresponding p-values. The Monte-Carlo exact test instead of the chi-square test was applied in the inferential analysis, if the proportion of cells with expected conjoint frequencies lower then 5 was more than 20%.

Multivariable Poisson models were performed to assess the effects of covariates on the probability of taking triptans, and results were expressed in terms of prevalence ratios (PRR), which are a better estimate of risk than odds ratios when the prevalence of the outcome of interest is high, with related 95% confidence intervals (95%CI) [26]. The covariates were introduced into the model through a stepwise procedure at a 0.2 significance level. Spearman correlation coefficients and p-values were evaluated to avoid multi-collinearity when implementing multivariable models. Statistical analyses were carried out using the SAS software (version 9.2).

### Ethics statement

The study was approved by the National Association of Headache Patients. The study was also approved by the Federation of the Orders of Italian Pharmacists (FOFI).

The subjects participated in the study on a voluntary basis, and they were orally informed on the characteristics and the purpose of the study. The questionnaire was anonymous. Personal data were not collected and there is no way to trace back the answers to a specific responder. Enrolled subjects expressed their consent to participate in the study orally. No written consent has been produced to ensure anonymity for the participants.

Therefore, according to Italian legislation [27] on the protection of personal data no ethical approval was required. Moreover, in this article, the already published [9] data from the 2016–2017 study has been further analysed.

## Results

The respondents were 4,424 and 24.3% reported taking triptans as their usual treatment for migraines (Table 1). The use of triptans is more frequent among females than males (26.2% and 19.0% respectively); in the over-55 age group (27.4% of the total); in residents in the Northern Italy (25.1% against 20.1%), and among subjects in the secondary school or diploma- *ISCED2-3* level of education group (26.3% against 20.8%). Concerning the clinical characteristics, the use of triptans increased with the increasing value of ID-migraine (38% for definite migraine); the number of days with migraine (44.9% for those with more than 60 days with migraine in 3 months); the duration of the disorder (30.9% for patients with migraine for more than 10 years); and in subjects in care at a specialist headache centre (68.2%). The use of triptans is, moreover, significantly higher among patients who perceive their migraine as a medical condition (41.1%), and among those in the medication over-user category (43.3%)

**Table 1:** Characteristics of the study population.

**Characteristics**	**Categories**	**Triptans’ users (%)**		**N (Total 4,424)**	**p-value**	**Drugs used (%)**			**N**^1^ **(Total 3,266)**	**p-value**
		**No N = 3 349**	**Yes N = 1 075**			**Only NSAIDs N = 2.379**	**Only triptans N = 572**	**NSAIDs and triptans N = 275**		
Gender	Female	73.8	26.2	3, 265	<0.0001	71.4	19.3	9.3	2,358	<0.0001
	Male	81.0	19.0	1,159		80.2	13.4	6.4	868	
Age	<30	87.0	13.0	585	<0.0001	87.6	5.6	6.8	444	<0.0001
	30–54	74.5	25.5	2,816		72.9	18.3	8.8	2,082	
	>54	72.6	27.4	1,023		67.6	23.6	8.8	700	
Residence area	North	74.9	25.1	3,726	0.0059	72.6	18.4	9.0	2,650	0.0083
	Centre and South	79.8	20.2	698		78.8	14.8	6.4	576	
Educational level	ISCED0-1	79.2	20.8	221	0.0002	73.6	18.6	7.8	140	0.002
	ISCED2-3	73.7	26.3	2,791		71.6	19.6	8.8	2,028	
	ISCED4-6	79.2	20.8	1,412		78.0	17.0	8.0	1,058	
ID-migraine	other headaches	94.3	5.7	389	<0.0001	94.4	3.0	2.6	305	<0.0001
	unlikely migraine	89.6	10.4	898		89.4	7.4	3.2	688	
	probable migraine	79.1	20.9	1,372		77.8	14.5	7.7	1,040	
	definite migraine	61.9	38.1	1,765		55.9	30.3	13.8	1,193	
**Characteristics**	**Categories**	**Triptans’ users (%)**		**N (Total 4,424)**	**p-value**	**Drugs used (%)**			**N**^2^ **(Total 3,266)**	**p-value**
		**No N = 3 349**	**Yes N = 1 075**			**Only NSAIDs N = 2.379**	**Only triptans N = 572**	**NSAIDs and triptans N = 275**		
Number of days with headache in the last three months	<11	82.2	17.8	3,024	<0.0001	81.1	12.7	6.2	2,250	<0.0001
	11–29	65.0	35.0	815		61.3	27.6	11.1	597	
	30–60	58.0	42.0	407		52.0	30.5	17.6	279	
	>60	55.1	44.9	178		43.0	36.0	21.0	100	
Number of days with headache in the last three months	≥45	55.9	44.1	320	<0.0001	46.4	34.2	19.4	196	<0.0001
Number of years with headache	<1	98.1	1.9	319	<0.0001	98.8	0.4	0.8	247	<0.0001
	1–4	84.2	15.8	805		84.1	11.0	4.9	599	
	5–9	80.2	19.8	698		79.1	12.4	8.5	507	
	≥10	69.1	30.9	2,602		65.7	23.6	10.7	1,873	
Who takes care of the patients^3^	Nobody	96.0	4.0	2,223	<0.0001	96.4	1.9	1.7	1,748	<0.0001
	General practitioner	65.1	34.9	1,348	<0.0001	59.3	24.4	16.3	896	<0.0001
	Specialist	44.3	55.7	668	<0.0001	32.0	49.8	18.2	440	<0.0001
	Headache centre	31.8	68.2	447	<0.0001	22.5	53.7	23.8	294	<0.0001
The headache is a disease	Yes	58.9	41.1	2,202	<0.0001	53.1	31.5	15.4	1,484	<0.0001
	No	92.3	7.6	2,222						
Medication Over-users	Yes	56.7	43.3	786	<0.0001	50.7	31.2	18.1	513	<0.0001
	No	79.8	20.2	3,638						
**Characteristics**	**Categories**	**Triptans’ users (%)**		**N (Total 4,424)**	**p-value**	**Drugs used (%)**			**N**^4^ **(Total 3,266)**	**p-value**
		**No N = 3 349**	**Yes N = 1 075**			**Only NSAIDs N = 2.379**	**Only triptans N = 572**	**NSAIDs and triptans N = 275**		
Pain medication solves the headache	Yes	83.5	16.5	1,997	<0.0001	81.9	14.0	4.1	1,578	<0.0001
	Often	76.2	23.8	1,415		74.0	14.8	11.2	990	
	Rarely	65.3	34.7	666		63.1	19.6	17.3	423	
	No	48.6	51.4	346		36.6	51.9	11.5	235	
Pain medication solves the headache Subgroup ID-migraine: other headaches n = 389	Yes	95.3	4.7	279	<0.06					
	Often	94.7	5.3	75						
	Rarely	88.9	11.1	27						
	No	75.0	25.0	8						
Pain medication solves the headache Subgroup ID-migraine: unlikely migraine n = 898	Yes	93.1	6.9	548	<0.0001					
	Often	90.0	10.0	240						
	Rarely	73.4	26.6	64						
	No	69.6	30.4	46						
Pain medication solves the headache Subgroup ID-migraine: probable migraine n = 1372	Yes	83.2	16.8	647	<0.0001					
	Often	82.0	18.0	456						
	Rarely	69.5	30.5	177						
	No	54.4	45.5	92						
Pain medication solves the headache Subgroup ID-migraine: definite migraine n = 1765	Yes	67.7	32.3	523	<0.0001					
	Often	64.8	35.2	644						
	Rarely	60.6	39.4	398						
	No	40.0	60.0	200						

^1^ Subjects taking other medicines or different combinations are not included.

^2^ Subjects taking other medicines or different combinations are not included.

^3^ More than one possible answer

^4^ Subjects taking other medicines or different combinations are not included.

The use of triptans for the treatment of migraine rises in percentage terms with the decrease in the perceived efficacy of pain medication: the highest percentage of triptan use (51.4%) was among those respondents that reported the inefficacy of the pain relief product being used; in confirmation of this fact, the majority (83.5%) of those that reported a beneficial effect from the pain relief medication being used declared that they did not use triptans. This pattern is confirmed in repeating the evaluation for the individual sub-groups of ID-migraine (Table 1).

All of the differences found are statistically significant (p-value<0,05).

The respondents treating headaches with triptans and/or NSAIDs were categorised in three groups: subjects using triptans only, subjects using NSAIDs only, and subjects using a combination of both medications (Table 1). We excluded subjects who took other combinations of medications for HDs. The majority of the respondents stated that they used NSAIDs only (73.8%), with the exception of those that reported no benefit from using pain medication, and subjects in the care of a specialist or a headache centre.17.7% of respondents use triptans only for treatment: this figure was higher among females; in the older age groups; among residents in the Northern Italy; those with a secondary school or diploma level of education- *ISCED2-3*; those with definite migraine, long duration, or with a high number of days with headache. Similar results are observed for subjects that associate triptans with NSAIDs for treatment (8.5%). The number of subjects in care with a specialist or a headache centre is higher among the triptan-only group, or using a combined therapy with NSAIDs.

Table 2 reports the prevalence ratios for the factors significantly associated with use of triptans (alone or in combination) that resulted from the multivariable analysis following a stepwise procedure. The use of triptans is directly associated with ID-migraine, being greater among those with probable or definite migraine. It is also more frequent among older patients, patients in care with a specialist or a headache centre, and patients with a longstanding condition or a higher number of days with headache, although without a clear increasing trend.

**Table 2:** Use of triptans and associated factors: Prevelence Ratios (PR) and 95% Confience Intervals (95% CI).

Covariate	Variable	PRR	95% CI
ID-migraine	other headaches	baseline	
	unlikely migraine	1.50	0.94–2.39
	probable migraine	2.06	1.33–3.19
	definite migraine	2.59	1.69–3.97
Age class	<30	0.73	0.56–0.94
	30–54	1.04	0.91–1.20
	>54	baseline	
Who takes care of the patients^5^	Nobody	0.13	0.10–0.18
	General practitioner	0.84	0.70–1.00
	Specialist	1.31	1.10–1.57
	Headache centre	1.54	1.29–1.84
Number of years with headache	<1	baseline	
	1–4	5.26	2.31–11.94
	5–9	4.84	2.13–10.98
	≥10	5.77	2.57–12.94
Number of days with headache in the last three months	<11	baseline	
	11–29	1.26	1.09–1.46
	30–60	1.28	1.07–1.52
	>60	1.36	1.07–1.73

^5^ For this question multiple answers were possible

To further control for potential confounders related to the different types of migraine, we limited the analysis to 3,137 subjects (70.9% of the total) classified as “definite” or “probable” migraines. The above associations are mostly confirmed (Table 3), although confidence intervals are generally wider due to the smaller numbers; only age appears no longer associated with the use of triptans in this subgroup of patients.

**Table 3:** Use of triptans and associated factors in subjects with definite or probable migraine: Prevalence Ratios (PR) and 95% Confidence Intervals (95% CI).

Covariate	Categories	PR	95% CI
Age class	<30	0.81	0.61–1.06
	30–54	1.09	0.94–1.27
	>54	baseline	
Who takes care of the patients^6^	Nobody	0.15	0.11–0.20
	General practitioner	0.84	0.70–1.01
	Specialist	1.33	1.10–1.60
	Headache centre	1.50	1.24–1.81
Number of years with headache	<1	baseline	
	1–4	4.46	1.81–10.95
	5–9	4.28	1.75–10.46
	≥10	5.16	2.14–12.47
Number of days with headache in the last three months	<11	baseline	
	11–29	1.18	0.98–1.42
	30–60	1.20	1.03–1.40
	>60	1.33	1.02–1.71

^6^ For this question multiple answers were possible

## Discussion

Over the last thirty years, triptans have gradually made their way among the therapeutic tools for combating migraine attacks. These drugs, agonists of the 5-HT 1B/1D receptor, are still considered the best pharmacological approach possible for managing the pain and symptomology that accompany the acute phase of a migraine [28, 29]. We are still awaiting new therapies such as ditans [30] and gepants [31], which have yet to prove their greater efficacy.

In theory, triptans, thanks to their selective vasorestrictive action, control of various neurotransmitters, among which the calcitonin gene related peptide, and the stabilisation of the sensitive endings of the trigeminovascular system, should have been extremely efficacious and were considered so, at least, in initial studies. They being an entirely new pharmacological class, it took some time before the medical profession became aware of their potential use, their efficacy, and also their substantial safety. The first to exploit these properties of triptans were specialists who routinely treat patients suffering from migraine. Only much later, and not uniformly, family doctors started their use. This explains why, as our data reveal, the higher the level of awareness of the product, or at least knowledge of the pathology, the more common their use is. Hence, the percentage of users is far greater among patients in care with a headache centre (68.2%) compared with those patients in care with a family doctor (34.9%). This is also evident when a patient perceives the headache as a medical condition (42.1%) compared with those who are unaware of this fact (7.6%). Furthermore, while triptans remain the gold standard for the acute treatment of migraine pain, it is also important when attempting to understand the use of this molecule to consider the gravity of the pathology: a higher score in the ID-migraine test, a prolonged duration of the disorder, a higher frequency of headache days per month are all important factors. The patient’s sex is also fundamental in that the greater prevalence of headache disorder among females means that female patients are more commonly using this therapy compared with males.

While expertise of the pathology and the specific therapeutic products for its treatment, as well as the seriousness of the migraine itself, are undeniably important factors, an explanation for the extremely low rates of triptan use compared to the large number of migraineurs must be sought elsewhere. Several studies have demonstrated that the use of triptans by migraineurs, despite the prevalence of the condition among the population, is actually much less common that could be expected. Concerning Italy in particular, a study of pharmacy-use [32] conducted on a large sample of the national population (5.57 million subjects, a tenth of the total population) found that approximately 40% to 60% of triptan users had received a single prescription in one year. Furthermore, a study carried out by Ifergane et al [33] found that among subjects that had received more than one prescription in one year, only 14.3% had tried a second type of triptan, and, of these, 52.1% had received just one prescription for the new triptan. These data were confirmed by a real-life analysis [34] conducted on 10,270,683 adults in which the authors concluded that unsatisfied therapeutic expectations regarded a large part of the patients treated with triptans. Therefore, it seems evident that there is a lack of virtuosity in the prescription procedure, given that the low usage of triptans in Italy cannot be ascribed to a cost factor for patients in that triptans are available free of charge through the National Health Service.

Further evidence of this comes from a recent Danish nationwide register-based cohort study focused on all residents with access to public healthcare in which complete purchase records for Sumatriptan, Naratriptan, Zolmitriptan, Rizatriptan, Almotriptan, Eletriptan, and Frovatriptan were evaluated over a 25-year period (January 1994 –October 2019). Even though the yearly prevalence of triptan use increased over time from 5.17 to 14.57 per 1000 inhabitants, only 12.3% of the Danish migraine population purchased a triptan. After the first purchase of a triptan, 43% of patients had not repurchased triptans within 5 years. The Authors concluded that they observed “low rates of triptan adherence, likely due to disappointing efficacy and/or unpleasant side effects rather than economic considerations” [35].

Ruling out financial factors on the part of the patient, but keeping in mind that this is an important factor the National Health Service in that it bears the costs of triptan therapy, it would be useful to perform a well-designed study in order to permit a thorough evaluation of the reasons behind the low adherence to therapy by patients.

It is necessary to this end to investigate whether, after the first course of triptans, the patient does not request another prescription from their family doctor, or actually refuses it if proposed again. In addition, it is important to understand whether this low adherence is linked to a perception of poor efficacy of the medicine, perhaps due to incorrect usage e.g., taking the triptan after the onset of the migraine, or adverse reactions of particular importance which, since the patient’s doctor is not informed, cannot be recorded, and hence correctly evaluated in terms of pharma vigilance. Finally, it is not currently known whether the reduced prescription of triptans is attributable to lack of trust or inadequate knowledge of these molecules on the part of the medical profession, in particular among family doctors.

## Limitations

Before proceeding with the administration of the questionnaire, the pharmacist asked the respondent to confirm that he or she did not participate in the survey before and / or in another pharmacy. However, as these are anonymous questionnaires, this event cannot be totally excluded.

Moreover, our work has some limitations due to the impossibility of collecting in-depth clinical data on the respondents. These limitations are directly related to the survey setting: our aim was to collect, thanks to community pharmacies, data on a large number of headache patients throughout an entire nation. It was therefore not possible to have a diagnostic definition of migraine according to the latest criteria of the International Headache Society, nor to achieve more information on the sleep hygiene or on the correctness of lifestyles. Furthermore, it was not possible to obtain precise information on the individual triptans taken, on the times and methods of their intake, on their effectiveness in a single attack and on the need to take a second dose or a rescue medication in case of ineffectiveness. All this information would undoubtedly be of considerable interest and suggest the idea of a future work carried out in a different setting and with the direct participation of headache clinicians in the data collection.

## Conclusions

Only 25% of patients suffering from HDs are prescribed triptans. Older patients, those with definite migraines, and those with a chronic disorder resort more frequently to this class of pharmaceuticals, as do those patients in care at a specialist headache centre. While the data available from the analysis of this study is limited compared with other published work in scientific literature, it has two clear advantages: first, allowing the performance of direct detection, in real life, on patients requesting pharmacological treatment for a migraine headache in progress and, secondly, not being based on data of usage of triptans derived from records of consumption of such medicines. This study also confirmed contemporarily with the ID-Migraine test the real presence in individual patients of forms of migraine, definite or probable as they may be.

Equally important was using community pharmacies as a point of clinical data gathering. Our study was performed in community pharmacies also with the aim of carrying out an epidemiological survey on large numbers. In this sense, community pharmacies are an excellent tool as they are distributed throughout the entire Italian national territory and have proven extremely effective over the years for the gathering of epidemiological data for the prevention of pathologies with a high incidence, as well as taking into care patients affected by chronic pathologies [9, 10–14].

However, this study only allows us to take a snapshot in a particular instant, a moment of need, when the patient enters the pharmacy to request treatment for headache. Repeating on the same patients several consecutive readings, would have allowed to confirm the real adherence to the various therapies of the acute attack not only on the basis of the patients’ recollection but on the basis of actual consumption data.

Any future studies should take advantage of community pharmacies, plan actions that would allow a series of evaluations over time of the requirements of migraineurs, and establish a process to put these patients under the care of the pharmacy to ensure adherence to therapy.

## Supporting information

S1 Questionnaire (DOCX)

S1 Database (XLS)

## Abbreviations

CH: Cluster headaches
FICEF Onlus: Italian Headache Foundation
FOFI: Federation of the Orders of Italian Pharmacists
HD: Headache Disorders
ID-MS: ID Migraine screener test
ISCED: International Standard Classification of Education
MOH: Medication-Overuse Headache
MOs: Medication Over-users
NSAIDs: Non-steroidal anti-inflammatory drugs
PH: Primary headache
PRR: Prevalence rate ratio
YLDs: Years Lived with Disability
