# Supplementary figures and images for: Triptans utilization in Italian population: A real-life study in community pharmacies
Source: PLoS One. 2023 Sep 8;18(9):e0291323. doi: 10.1371/journal.pone.0291323 (PMC10490971; doi:10.1371/journal.pone.0291323)

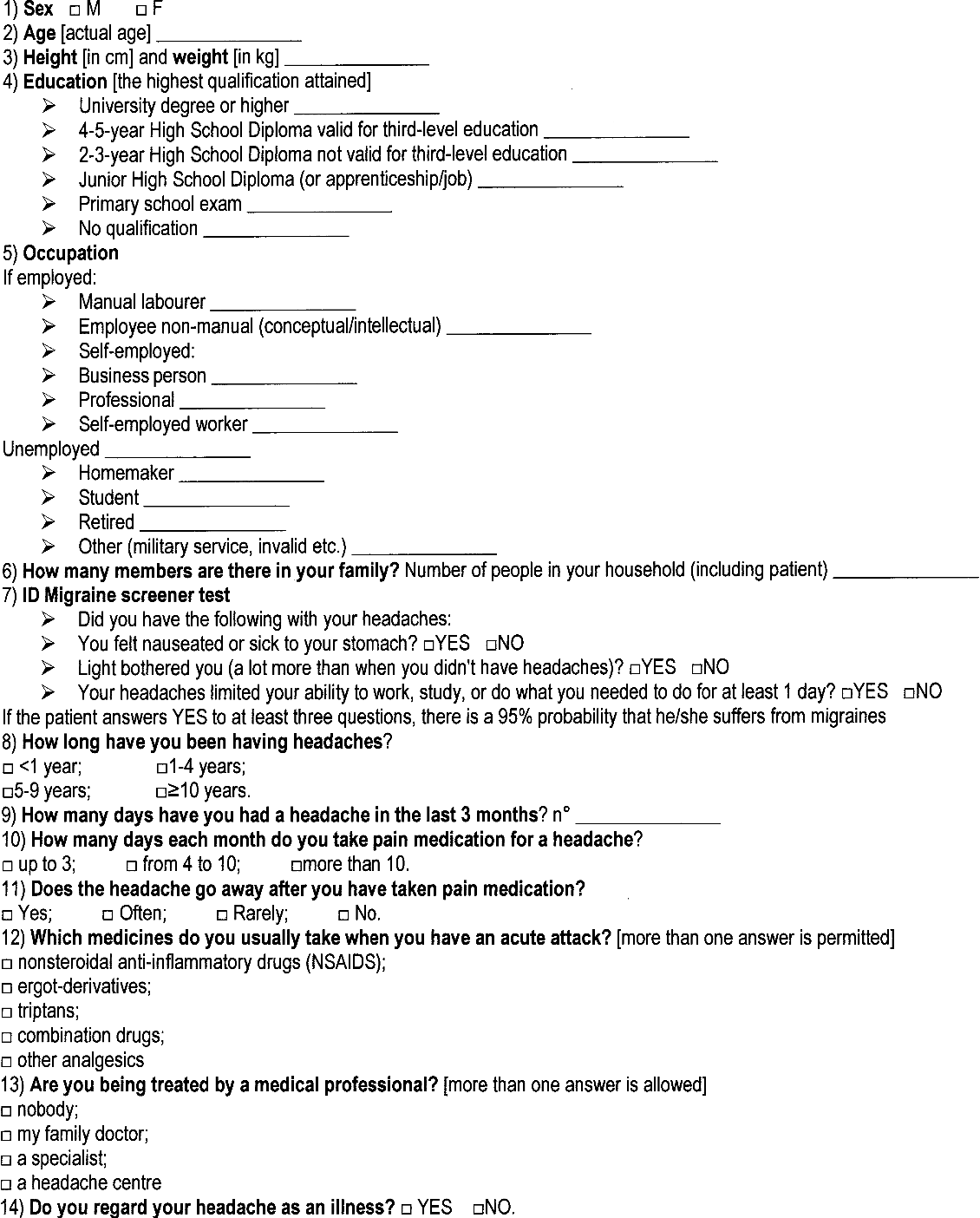


Fig.1 Questionnaire administered to the surveyed pharmacy users.

Supplement: S1 Questionnaire — (DOCX) [file pone.0291323.s001.docx]
